# Abundance, diversity and richness of natural enemies of the fall armyworm, *Spodoptera frugiperda* (J.E. Smith) (Lepidoptera: Noctuidae), in Zambia

**DOI:** 10.3389/finsc.2023.1091084

**Published:** 2023-10-20

**Authors:** Gilson Chipabika, Philemon H. Sohati, Fathiya Mbarak Khamis, Patrick C. Chikoti, Robert Copeland, Levi Ombura, Paul W. Kachapulula, Tamara K. Tonga, Saliou Niassy, Subramanian Sevgan

**Affiliations:** ^1^ School of Agricultural Sciences, Department of Plant Science, University of Zambia, Lusaka, Zambia; ^2^ Department of Plant health, International Centre of Insect Physiology and Ecology, Nairobi, Kenya; ^3^ Plant Protection Division, Zambia Agriculture Research Institute, Mount Makulu Research Station, Lusaka, Zambia

**Keywords:** fall armyworm, agroecological region, parasitoids, predators, biological control, Zambia

## Abstract

The fall armyworm (FAW), *Spodoptera frugiperda*, an invasive pest originating from the Americas is a serious pest threatening cereal production and food security in Zambia. We studied the prevalence and abundance of natural enemies of FAW in three Agroecological regions (AERs I, II, and III) to identify those that could potentially serve as bio-control agents. Sampling of FAW parasitoids and predators was done along trunk roads at intervals of 10 km. Molecular sequence analysis and morphological characterization were used to identify natural enemies. Over 11 species of FAW natural enemies, including egg, egg-larval, and larval parasitoids, and predators, were identified in Zambia. The mean number of natural enemies and species richness was higher in AER I and IIa. Consequently, egg parasitism was highest in those two regions, at 24.5% and 12.2%, respectively. Larvae parasitism was highest in AER I (4.8%) and AER III (1.9), although no significant differences were observed. The most abundant and widely distributed parasitoid was *Drino* sp. (Diptera: Tachinidae), while *Rhynocoris segmentarius* (Germar) (Hemiptera: Reduviidae) and *Belanogaster* sp. (Hymenoptera: Vespidae) were the most prevalent predators. Our study reveals the presence of two natural enemies belonging to the genus *Tiphia* and *Micromeriella*, uncommon to FAW. Significant differences in the number of parasitoids were observed in polycropping, with the highest recovery of 12 ± 10% from maize + cowpeas + pumpkin and watermelon mixed cropping. The higher the rainfall, the lower the number of natural enemies recorded. Variations in rainfall patterns which affect FAW availability, cropping systems and the three AERs may explain natural enemies’ species diversity in Zambia. The information provided in this study can aid the development of a national biological control programme for sustainable management of fall armyworm.

## 1 Introduction

In Sub-Saharan Africa (SSA), maize is the most important crop (1), but its productivity is constrained by many lepidopteran insect pests such as stemborers (2). The arrival of fall armyworm (FAW) (*Spodoptera frugiperda*, J. E.Smith (Insecta: Lepidoptera: Noctuidae), exacerbated the food security threat at household and national levels in Zambia. FAW is an invasive species that originated in sub-tropical and tropical regions of the Americas (3, 4). FAW causes significant maize yield losses, ranging from 11.5 to 73% under severe infestation (5, 6). In Brazil alone, costs to control the FAW in maize have exceeded 600 million USD annually (7). The pest is polyphagous and has approximately 353 host plant species (8), including cultivated crops (9, 10), however, it prefers plants from the family Poaceae, maize especially. The female produces up to 2000 eggs and can have 10–12 generations per year (11). In Africa, the FAW was first reported in January 2016 in Nigeria (12, 13). In Zambia, the pest was reported in Chirundu in November of the same year, from where it spread to all ten provinces, infesting and causing damage to maize within three months (14).

The management of *Spodoptera* species in Zambia is mainly through use of synthetic pesticides. The Government of the Republic of Zambia adopted emergency measures and spent over $3million on the procurement of insecticides, personal protective suits, equipment, early maturing maize varieties, and distribution of requisites to various districts in one year (15, 16). However, synthetic pesticides are generally non-selective, hazardous to the applicator, and highly toxic to natural enemies of the target species (17–19).

Control strategies, such as push–pull (20), maize-legume intercropping (21), microbial control (22), and augmentative biological control strategies (23), are being developed and implemented for the management of FAW. These strategies enhance crop performance and ecosystem services, such as the regulation of pest populations by natural enemies (24). Therefore, the sustainable management of FAW requires an integrated approach that is mindful of natural processes.

Natural enemies, especially parasitoids and predators, are critical components of Integrated Pest Management (IPM). IPM is considered a more sustainable approach for pest control as it relies on the most economical means and with the least hazard to humans and the environment. Although FAW is an invasive pest in Africa, several new associations of the pest with natural enemies already prevalent in Africa have been reported from East Africa (25). Regarding Southern Africa, Durocher-Granger et al. (26) reported the presence in Zambia of *Metopius discolour* Tosquinet, *Chelonus bifoveolatus* Szépligeti (Hymenoptera: Braconidae), *Coccygidium luteum* Brullé (Hymenoptera: Braconidae), *Chelonus curvimaculatus* Cameron (Hymenoptera: Braconidae), *Drino quadrizonula* Thomson (Diptera: Tachinidae) *Charops* sp., *Cotesia icipe* Fernández-Triana and Fiaboe (Hymenoptera: Braconidae), *Euplectrus laphygmae* Ferrière (Hymenoptera: Eulophidae), *Parapanteles* sp., *Diadegma* sp., *Pristomerus* sp. and *Enicospilus capensis* Thunberg (Hymenoptera: Ichneumonoidea). In Mozambique, Caniço et al. (27) reported the occurrence of *C. luteum*, *Charops* sp., *Metopius cf. discolour* Tosquinet (Hymenoptera: Ichneumonidae) and *D. quadrizonula.* Understanding the abundance and diversity of indigenous FAW natural enemies could facilitate the development of augmentative and conservation biological control programs in Zambia.

Surveys conducted in four locations in Lusaka and Central provinces in Zambia highlighted the new association of existing natural enemies of FAW (26). A more comprehensive assessment of FAW natural enemies in diverse Agroecological regions (AERs) in Zambia is of paramount importance for developing and implementing a robust IPM package to minimize the misuse of chemical pesticides and protect food security. Therefore, the objective of the study was to assess the abundance and diversity of indigenous natural enemies of FAW in AERs I, IIa and III of Zambia and to identify candidates that would serve as biological control agents.

## 2 Materials and methods

### 2.1 Study area

**Figure 1:**
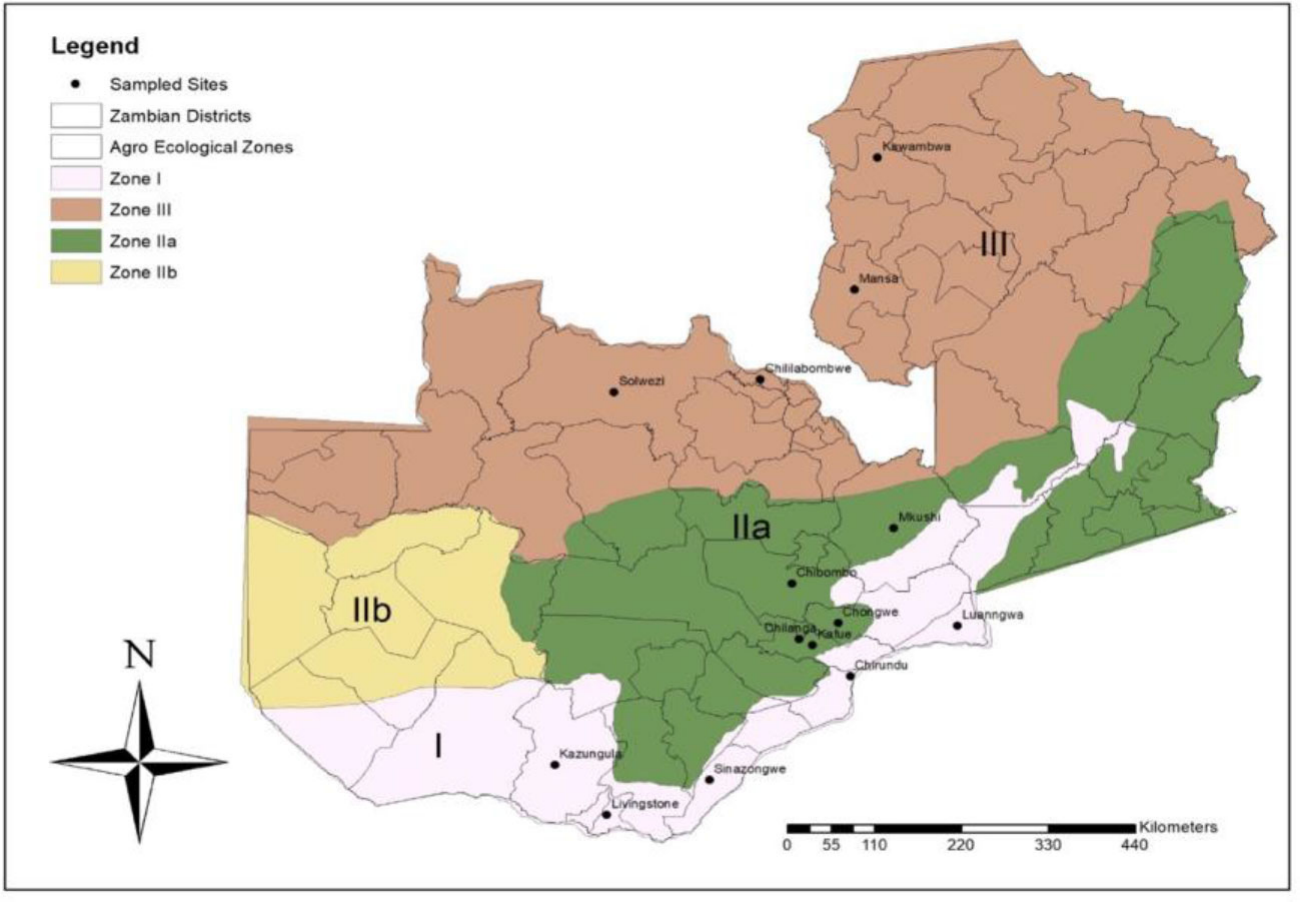
Map showing Agroecological regions and districts surveyed for natural enemies of fall armyworm in Zambia between March and May 2019.

Zambia is divided into three mains AERs, and the survey for the natural enemies of FAW was conducted in AERs I, IIa and III (**Figure 1** and **Table 1**). The areas and sites in all the AERs were selected based on reports of FAW occurrence (15). Agroecological region I receives below 800 mm of rainfall per year, with mean daily temperatures ranging from 20 **°**C to 25 **°**C during the rainy season and up to 38 **°**C in the dry, hot season. Agroecological region II experiences rainfall ranging from 800 to 1000 mm and a mean daily temperature range from 20 to 23°C up to 25 **°**C during the rainy season, and AER III receives between 1000 and 1500 mm annually with a mean daily temperature of 16 **°**C in the rainy season and 18 **°**C in the dry season (28).

**Table 1:** Climatic data for the three Agroecological regions of Zambia in 2019.

Agroecological region	Coordinates	District	Altitude	Average temperature (°c)	Rainfall (mm)	Rain days
I	1526.542 S 3012.962E	Luangwa	366	28.9	771.5	78
I	1557.269 S 2854.063E	Chirundu	376	31.7	164	x
I	1704.442 S 2720.302E	Sinazongwe	865	31.5	681.2	16
I	1751.48 S 2533.799E	Livingstone	915	30.6	410.9	x
I	1737.259 S 2550.261E	Kazungula	1044	x^1^	623	x
IIa	1524.21 S 2834.000E	Chilanga	1138	22.7	464.5	49
IIa	1536.606 S 2816.849E	Kafue	1144	x	740.3	66
IIa	1513.892 S 2831.485E	Chongwe	1142	21.7	380.4	46
IIa	1443.694 S 2804.775E	Chibombo	1173	21.5	710	44
IIa	1332.056 S 2933.212E	Mkushi	1446	20.6	947.8	69
III	1213.332 S 2618.332 E	Solwezi	1384	x	1109	56
III	1220.138 S 2751.625E	Chililabombwe	1312	x	1049.6	72
III	1108 S 2754.231E	Mansa	1220	x	750	106
III	1220.138 S 2750.320 E	Kawambwa	1382	x	1368.3	131
III	0945.882 S 2849.590 E	Mwansabombwe	1381	x	1300	130

x1 = data not available.

Source, Zambia Meteorological Department, 2019.

In AER I, Luangwa, Chirundu, and Sinazongwe have loamy and clay soils, with coarse to fine loam top soils that are slightly acidic to alkaline. The soils have good soil moisture holding characteristics. Kazungula and Livingstone have reddish course soils with medium acidity. The main vegetation in this region is Mopani, with an open deciduous canopy. It is dominated by *Colophospermum mopane* (28, 29) (**Table 1**).

Agroecological region II is dominated by sandy, acidic soils that have low nutrient reserves and poor water retention when compared to AER I. These soils dry up easily during dry spells and are prone to leaching in the event of heavy rainfall. Agroecological region II is subdivided into sub-region IIa comprising of the degradation Sandveld plateau, and sub-region IIb comprising the degradation Kalahari sand plateau and Zambezi flood plain in the Western province (30). The vegetation in AER II is dominated by miombo woodland, where the *Acacia combretum* and *Acacia terminalia* are the most prevalent tree species in Lusaka, Central, Eastern, and Southern provinces (28) (**Table 1**).

Soils in AER III are highly weathered, leached, and highly acidic. They have limited plant nutrients to support plant growth but have high biological activity. The vegetation in the region is miombo and a mixture of Chipya and dry evergreen forest (28) (**Table 1**).

### 2.2 Assessment of natural enemies of fall armyworm

A survey of natural enemies was conducted in 25 maize fields in AER I, 41 in AER IIa, and 24 in AER III during the late rainy season of maize production from March to May 2019 (**Table 2**). A minimum of four districts in each AER and five fields per district were sampled. Geolocational data, latitude, longitude, and altitude were taken using GPS (GARMIN: GPS MAPR 78s). Sampling was conducted along main trunk roads at intervals of 10 km on maize and sorghum fields. In each field, 50 plants were examined visually for the presence of FAW and natural enemies (including their egg batches, larva and pupa), following along a ‘W’ configuration transect. Maize and sorghum plants were sampled from the vegetative stage to maturity (31, 32).

**Table 2:** Incidence of Fall armyworm eggs, larvae, parasitoids, and predators collected from fields in Agroecological regions I, IIa and III of Zambia in 2019.

Agroecological region		I	IIa	III	Total
Sites surveyed		25	41	24	90
	Egg batches collected	40	55	10	105
	Total larvae collected	863	1520	550	2933
Dead larvae during	Transportation	25	23	27	75
	Larvae survival during collection and rearing	838	1497	523	2858
Egg parasitoid species	*Trichogramma* sp	0	1	0	1
	*Telenomus* sp	1	0	0	1
Egg larvae parasitoid species	*Chelonus* sp	9	2	0	11
Larval parasitoids species	*Cotesia* sp	0	1	0	1
	*Tiphia* sp	2	0	0	2
	*Coccygiduim luteum*	3	5	2	10
	*Drino* sp	20	4	1	25
	*Drino quadrizonula*	6	12	0	18
	Unidentified Tachinid sp	7	0	3	10
	*Micromeriella sp*	0	2	0	2
	*Charops* sp	9	2	0	11
Predator species					
Heteroptera: Pentatomidae	*Glypsus conspicuous*	5	3	0	8
Hymenoptera Vespidae	*Belanogaster* sp	20	13	5	38
Heteroptera: Reduviidae	*Rhynocoris segmentari*	25	21	1	47

Each maize plant was inspected for the presence of natural enemies predating on the FAW egg batches and larvae. The FAW natural enemies were collected directly (predators observed preying on the host) or indirectly (collection of FAW larvae and egg batches from which some parasitoids emerged) from the field. Inspections were done on the middle leaves where egg batches are laid, the upper new leaves, and the whorl where FAW larvae feed and hide.

The eggs were collected by cutting out the leaf tissue with the egg batch from the maize or sorghum plant to avoid dislodging them. The samples were then placed in a petri dish with a piece of paper towel inserted at the base to keep the egg batch and leaf tissue moist. The collection of predators was done simultaneously with FAW eggs and larvae.

Predators seen attacking or feeding on larvae were collected using aspirators and forceps and were put in one-liter jars lined with moistened tissue paper. Some of them were placed in vials with 70% ethanol for preservation. All stages of FAW were collected, and each larvae was placed in a petri dish (9 cm diameter) containing fresh pieces of leaves and stalks as a food source. Before placing the larvae in the petri dish, a piece of tissue paper was laid at the bottom to absorb excess moisture from larvae’s frass and natural diet (replaced every 48 hours). A lid was then placed on top and loosely tied with a rubber band to prevent larvae or parasitoids from escaping.

The Petri dishes were then placed in a thermo-electrical cooler (Campingazi thermoelectric system 12v # 08120OTE 016776) and plugged into the car and delivered to Mt. Makulu Research Station, Entomology laboratory in Chilanga. The larvae were reared on artificial diet (General Diet for Lepidoptera, Product Number F9772, with antibiotic of 14% active chlortetracycline from Frontier TM Agricultural Sciences, Network, DE, USA) in the laboratory. The newly hatched larvae were reared in vials on artificial diet for at least 10 days while observing for egg-larvae parasitoid emergence using a hand lens. Monitoring for parasitoid emergence was done daily in the laboratory.

### 2.3 Identification of natural enemies

The genomic DNA of adult parasitoids was extracted from the hind legs of the individual insects by using the Isolate II Genomic DNA Kit (Bioline, London, United Kingdom), following the manufacturer’s instructions. The resultant DNA was eluted in a final 50 μl volume, and the quality and quantity checks were done using the Nanodrop 2000 Spectrophotometer (Thermo Fischer Scientific, Wilmington, USA).

The mitochondrial COI gene and the ribosomal domain 2 (D2) region of 28S rDNA were amplified through Polymerase Chain Reaction (PCR) using standard primers LepF1 (5’-ATTCAACCAATCATAAAGATATTGG-3’) and LepR1 (5’ –TAAACTTCTGGATGTCCAAAAAATCA- 3’) (33) for the COI gene, and LepD2F (5’- AGTCGTGTTGCTTGATAGTGCAG- 3’) and LepD2R (5’- TTGGTCCGTGTTTCAAGACGGG- 3’) (34, 35) for the D2 region of 28S rDNA. The PCR reactions were performed in total volumes of 20 µL using 5X My Taq Reaction Buffer (Bioline), 0.5 pmol µL-1 of each primer, 0.5 mM MgCl2, 0.0625 U µL-1 My Taq DNA polymerase (Bioline), and 15 ng µL-1 of DNA template. The PCR cycling conditions set in an Eppendorf Mastercycler^®^ nexus gradient thermal cycler (Eppendorf, Germany) included an initial denaturation step at 95°C for 2 min, followed by 40 cycles of 30 sec at 95°C, 30-sec annealing (52°C for LepF1/R1 and 58.8°C for LepD2 F/R), and 1 min at 72°C, with a final elongation step of 10 min at 72°C. The PCR products were separated on a 1.2% agarose gel, and the DNA bands were analysed and documented using a KETA GL imaging system trans-illuminator (Wealtec Corp, Meadowvale Way Sparks, Nevada, USA). The amplified products were purified using Isolate II PCR and Gel Kit (Bioline) and shipped to Macrogen Europe BV (Meibergreef, Amsterdam, the Netherlands), for bi-directional sequencing (using two primers to simultaneously sequence both strands of the PCR product). The successful sequences were assembled and edited using Geneious Version 8 (http://www.geneious.com) (36), generating a consensus sequence for each sample. From the consensus sequence, the forward and reverse primers were identified and removed. For conclusive identification of the species from both markers, similarity searches were conducted by querying the consensus sequences *via* BLASTn at the GenBank database hosted by the National Centre of Biotechnology Information (NCBI). BLAST (Basic Local Alignment Search Tool) algorithm finds regions of local similarity between sequences, in which consensus sequences were compared to publicly available sequences in GenBank. In addition to this, the query was also done in the Barcode of Life Database (BOLD). The molecular identification of fall armyworm natural enemies was later compared with the morphological identifications undertaken in the Biosystematics Unit, icipe.

### 2.4 Data analysis

The primer sequences were identified and removed from the consensus sequences generated from both the forward and reverse reads. For conclusive identification of the species from both markers, similarity searches were conducted by querying the consensus sequences *via* BLAST at the GenBank database hosted by the National Centre of Biotechnology Information (NCBI). The BLAST (Basic Local Alignment Search Tool) algorithm finds regions of local similarity between sequences, in which consensus sequences are compared with reference sequences in the GenBank database. In addition, the query was also done in BOLD (Barcode of Life Database).

The number of egg batches and larvae collected from AERs was recorded and analysed. Percent parasitism (% Parasitism) was calculated as follows: % Egg parasitism = (Number of egg batches parasitized/Total number of egg batches collected) x 100. Larval parasitism was calculated as follows: % Larval parasitism = (Number of live larvae parasitized (Nfp)/Total number of collected larvae (Nfc)) x 100 (27, 37, 38). Parasitoids emergence data from FAW collected in mono, poly and intercrops, as well as predators observed, was recorded.

The number of collected natural enemies was calculated by adding the number of parasitoids and predators and subjected to statistical analysis using R, version 3.6.1 (39). The mean number of egg batches, FAW larvae, % parasitism, number of parasitoids and predators per AER and natural enemies per cropping practice were compared using the Turkey test (P ≤ 0.05).

Regression analysis was used to test the influence of rainfall on the number of natural enemies across study areas. Relative abundance (RA) of the parasitoids and predators was determined by counting the number of individuals of a given parasitoid or predator species (Ni) divided by the total number of all individuals of all parasitoid or predator species (N) and converted to percent values (23). Parasitoid and predator diversity was determined by applying the Shannon–Weiner diversity index (H) (40).

## 3 Results

### 3.1 Identification of natural enemies

A total of 90 locations were surveyed across AERs I, IIa and III for the occurrence, abundance and diversity of FAW natural enemies in Zambia (**Table 2**). A total of 90 larvae parasitoids were recorded from the three AERs of Zambia, with AER I registering the highest (56), followed by AER IIa (26), and lastly AER III (6) (**Table 2**). The *Drino* species occurred in all three AERs and recorded the highest number of 25 individual specimens. It was followed by *Drino quadrizonula*, with 18 specimens, which occurred only in AER I and AER IIa. Other species recorded included 10 specimens of *Coccygiduim luteum* from all the AERs of Zambia, however, 11 specimens of *Chelonus* sp were obtained from AERs I and IIa. Three specimens of *Charops* sp were recorded from Kuzungula district in AER I. Two specimens each of *Tiphia* sp and *Micromeriella* sp were obtained from Livingstone and Chongwe districts in AERs I and IIa, respectively. There were also 10 specimens of unidentified Tachinidae species obtained from Luangwa (5) and Livingstone (2) districts in AER I, and in Kawambwa (1) and Mwansabombwe (2) districts in AER III (**Table 3**).

**Table 3:** Number of parasitoids of fall armyworm egg and larval found in maize fields from Agroecological Regions I, IIa and III of Zambia in 2019.

Type	Parasitoid	AER I						AER IIa						AER III					
		Luangwa	Chirundu	Kazungula	Livingstone	Sinazongwe	Total	Chibombo	Chongwe	Chilanga	Mkushi	Kafue	Total	Mansa	Kawambwa	Chililabombwe	Solwezi	Mwansabombwe	Total
Larval parasitoid	*Cotesia* sp	0	0	0	0	0	**0**	1	1	0	0	0	**2**	0	0	0	0	0	**0**
	Unidentified Tachinid sp	5	0	0	2	0	**7**	0	0	0	0	0	**0**	0	1	0	0	2	**3**
	*Coccydgium luteum*	0	3	0	0	0	**3**	0	4	1	0	0	**5**	0	0	2	0	0	**2**
	*Tiphia* sp	0	0	0	2	0	**2**	0	0	0	0	0	**0**	0	0	0	0	0	**0**
	*Drino* sp	0	2	18	0	0	**20**	2	2	0	0	0	**4**	0	0	0	0	1	**1**
	*Drino quadrizonula*	0	2	4	0	0	**6**	12	0	0	0	0	**12**	0	0	0	0	0	**0**
	*Micromeriella* sp	0	0	0	0	0	**0**	0	2	0	0	0	**2**	0	0	0	0	0	**0**
	*Charops* sp	0	0	3	0	0	**3**	0	0	0	0	0	**0**	0	0	0	0	0	**0**
Egg larval parasitoid	*Chelonus* sp	5	4	0	0	0	**9**	0	2	0	0	0	**2**	0	0	0	0	0	**0**
Egg parasitoid	*Telenomus* sp	0	0	0	0	1	**1**	0	0	0	0	0	**0**	0	0	0	0	0	**0**
	*Trichogramma* sp	0	0	0	0	0	**0**	0	0	1	0	0	**1**	0	0	0	0	0	**0**

Bold values indicate parasitoids identified in each Agroecological region.

The total number of predators collected were 93 and the highest number (19) was obtained from Chirundu district in AER I. This was followed by Sinazongwe district (13) in the same AER. Across the AER, AERI recorded the highest (50) predators followed by AER IIa (37) and lastly AERIII (6). The widely distributed predator species were *Belanogaster* and *Rhynocoris segmentarius*, which were recorded in all the three AERs (**Table 4**).

**Table 4:** Number of predators observed and collected from Agroecological regions I, IIa and III of Zambia in 2019.

Type	Predator	AER I						AER IIa						AER III					
		Luangwa	Chirundu	Kazungula	Livingstone	Sinazongwe	Total	Chibombo	Chongwe	Chilanga	Mkushi	Kafue	Total	Mansa	Kawambwa	Chililabombwe	Solwezi	Mwansabombwe	Total
Larval predator	Heteroptera: *Glypsus conspicuous*	0	4	0	1	0	**5**	0	1	1	0	1	**3**	0	0	0	0	0	**0**
	Hymenoptera: *Belanogaster*	2	6	3	1	8	**20**	2	2	4	0	5	**13**	0	5	0	0	0	**5**
	Heteroptera: *Rhynocoris segmentarius*	6	9	1	4	5	**25**	9	5	6	1	0	**21**	1	0	0	0	0	**1**
		8	19	4	6	13	**50**	11	8	11	1	6	**37**	1	5	0	0	0	**6**

Bold values indicate predators identified in each Agroecological region.

From the FAW egg batches and larvae collection, 2 egg parasitoid species emerged from egg batches, while 1 egg–larval parasitoid and 8 larval parasitoid species emerged from larvae. The egg parasitoids were obtained from AER I and AER IIa, while larval parasitoids were obtained from all the three AERs. A total of 3 predatory species predating on FAW larvae were observed.

Molecular characterization of the parasitoid targeting the 28S rDNA D2 gene region identified the parasitoid to the species level. The complete mitochondrial COI gene was obtained from whole genome sequencing, and this resolved the identity of the parasitoid up to species level. A BLAST search of the extracted COI gene generated from this study (GenBank accession number OR058595) had a 100% similarity with *Cotesia icipe* (MN900735.1). *Drino quadrizonula* constituted 20% of the total larvae parasitoids reared and was identical to *D. quadrizonula* (**Table 5**). This *Drino quadrizonula* was recovered from AER I and IIa. *Rhynocoris segmentarius* shared 100% identity with *R. segmentarius* sp. (GenBank FJ230538.1) (**Table 6**), and accounted for 50.5% of the larvae predator (**Table 4**). It was recovered from AER I, IIa, and III.

**Table 5:** Identification of parasitoids for FAW obtained in Zambia.

Parasitoid	Family	Location	Lep D2		Lep F1/R1		Method*
			Similarity (%) to GenBank sequence	Query %	Similarity (%) to GenBank sequence	Query %	
Egg parasitoid							
*Telenomus* sp.	Scelionidae	Sinazongwe	JX683253.1 (98.7)	86			MOL
*Trichogramma* sp.	Trichogrammatidae	Chilanga					MOR
Egg-larvae parasitoid							
*Chelonus* sp	Braconidae	Luangwa, Chirundu, Chongwe					MOR
*Chelonus* sp.	Braconidae	Chibombo	XR_004690329.1 (86.25)	100			MOL
Larvae parasitoid							
*Cotesia* sp.	Braconidae	Chongwe					MOR
*Cotesia icipe*	Braconidae	Chibombo	EU402134.1 (98.41)	93	MN900735.1 (99.68)	100	MOL
*Coccygidium luteum*	Braconidae	Chilanga, Chongwe, Chirundu, Chililabombwe					MOL
*Charops* sp.	Ichneumonidae	Chirundu					MOR
Unidentified Tiphiidae	Tiphiidae	Chirundu					MOR
*Drino* sp	Tachinidae	Luangwa, Chilanga,	EF183825.1 (93.36 – 95.07)	95 – 100	KY315738.1 (98.05 – 98.33)	91 -97	MOL
*Micromeriella* sp	Scoliidae						MOR
*Drino quadrizonula*	Tachinidae	Chibombo, Kazungula, Chirundu	EF183825.1 (95.07)	100	MN907776.1 (96.15)	99	MOL
Unidentified Tachinidae	Tachinidae	Kawambwa, Mwansabombwe, Livingston, Luangwa					MOR

* MOL, Molecular identification; MOR, Morphological identification.

**Table 6:** Identification of predators of FAW recovered from Zambia.

Predator	Family	Location	LepD2		Lep F1/R1		Method*
			Similarity (%) to GenBank sequence	Query %	Similarity (%) to GenBank sequence	Query %	
*Rhynocoris segmentarius*	*Reduviidae*	Chibombo Chongwe	FJ230538.1 (100)	100			MOL
Hymenoptera *Belanogaster* sp	Vespidae	Chibombo, Chirundu Livingstone, Chilanga Kawambwa, Chongwe Kazungula, Sinazongwe Kafue, Luangwa					MOR
Unidentified Reduviidae	*Reduviidae*	Chirundu, Luangwa Livingstone, Kazungula, Sinazongwe, Chibombo Chongwe, Chilanga Mkushi, Mansa					MOR

* MOL, Molecular identification; MOR, Morphological identification.

**Figure 2:**
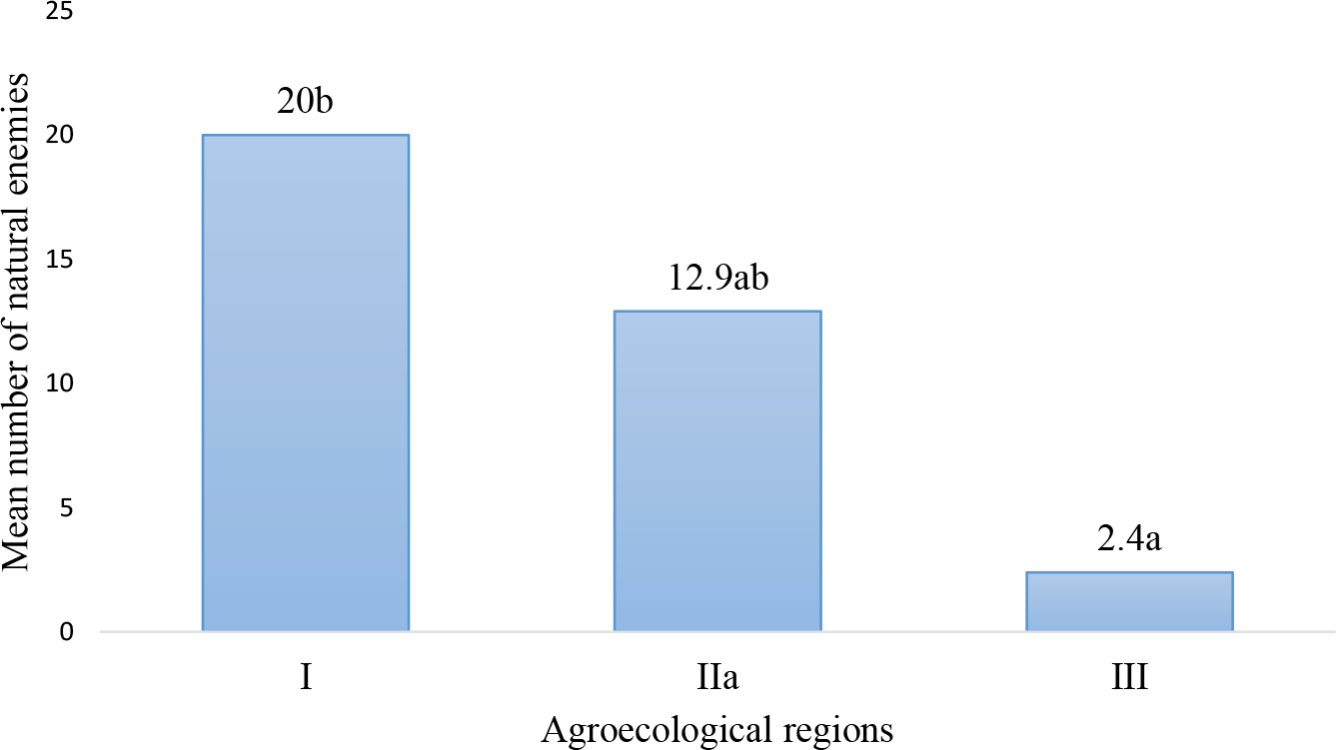
Mean number of natural enemies for fall armyworm recorded from Agroecological regions of Zambia.

The highest number of natural enemies was recorded from AER I and AER IIa, while AER III had the lowest (**Figure 2**). There were significant differences (degrees of freedom (df = 2; P = 0.01) in the occurrence of FAW natural enemies collected from AERs of Zambia.

### 3.2 Percent parasitism

#### 3.2.1 Percent egg and larval parasitism

There was a significant difference (P < 0.05) in the number of egg batches obtained from AER IIa, compared with AER I and III. However, egg parasitism was only recorded in AER I and IIa, while larval parasitism was observed in all regions, although there was no significant difference across them (**Table 7**).

**Table 7:** Mean percent parasitism of Fall armyworm egg batches and larvae in Agroecological regions I, IIa and III of Zambia in 2019.

AER	I	IIa	III
	Means ± SE	Means ± SE	Means ± SE
No. of egg batches	8.0 ± 1.8ab	11.0 ± 3.3b	2.0 ± 1.2ab
No. of larvae	182.8 ± 46.3a	315.2 ± 114.5a	112.8 ± 50a
% egg batch parasitism	24.5 ± 2.2a	12.2 ± 2.2a	0.0 ± 00a
% larvae parasitism	4.8 ± 1.6a	1.4 ± 1.9a	1.9 ± 0.9a

Means followed by the same letters horizontally are not statistically different according to Turkey P < 0.05.

#### 3.2.2 Number of fall armyworm natural enemies in maize mono and intercrops

During the survey, FAW larvae were collected from monocrop and intercrop fields. High numbers of natural enemies were collected from intercropped fields (df = 9; P = 0.01). The highest occurrence of natural enemies came from field’s intercroped with maize + cowpeas + pumpkin and watermelon (**Table 8**). The lowest number of natural enemies was recovered from maize mono crop.

**Table 8:** Mean number of fall armyworm natural enemies from maize mono and intercrops in Agroecological regions I, IIa and III of Zambia in 2019.

AER	Maize cropping system	Mean number of parasitoids	Mean number of predators	Mean number of natural enemies
		Mean ± SE	Mean ± SE	Mean ± SE
**I**	M + C + P	5.0 ± 4ab	4.0 ± 1a	9.0 ± 5a
**IIa**	M + W + P	7.0 ± 3ab	5.0 ± 0a	12.0 ± 4a
**III**	M + CS	0.0 + 0a	0.0 ± 0a	0.0 ± 0a
**III**	M + CS + B	1.0 ± 1ab	2.0 ± 1a	4.0 ± 2a
**IIa**	M + B	1.0 ± 1ab	6.0 ± 0a	6.0 ± 1a
**I, IIa**	M + C + P + W	12.0 ± 10b	1.0 ± 0a	14.0 ± 7a
**I, III**	M + P	3.0 ± 1ab	5.0 ± 1a	8.0 ± 4a
**IIa**	M + P + PP	5.0 ± 3ab	4.0 ± 2a	6.0 ± 5a
**I**	M + S	2.0 ± 1ab	3 ± 1a	5 ± 2a
**I, IIa, III**	Mm	0.0 ± 0a	2 ± 1a	2 ± 1a

AER, Agroecological region; Mm, Maize monocropping; B, Beans; C, Cowpeas; P, Pumpkins; W, watermelon; S, squash; CS, cassava; PP, Push-Pull. Means followed by the same letters vertically are not different statistically according to Turkey P ≤ 0.05.

#### 3.2.3 Abundance, diversity, and richness of fall armyworm parasitoids

FAW parasitoid species’ diversity and richness were highest in AER I, 0.2 and 8, respectively. This was followed by AER II with a diversity of 0.1 and richness of 7. AER III had the lowest diversity (0) and richness (4). Unidentified Tachinid sp were the most abundant (50%) in AER III followed by *Drino quadrizonula* (43%) in AER IIa (**Table 9**).

**Table 9:** Relative abundance, diversity, and richness of fall armyworm parasitoid species in Agroecological regions I, IIa and III in Zambia.

Parasitoid	Agroecological region					
	I		IIa		III	
	No. of individuals	Relative Abundance (%)	No. of individuals	Relative Abundance (%)	No. of individuals	Relative Abundance (%)
*Cotesia* sp	0	0	2	7	0	0
*Tiphia* sp	2	4	0	0	0	0
*Coccydgium luteum*	3	6	5	18	2	33
*Chelonus* sp	9	18	2	7	0	
*Drino* sp	20	39	4	14	1	17
*Drino quadrizonula*	6	12	12	43	0	0
Unidentified Tachinid sp	7	14	0	0	3	50.0
*Micromeriella* sp	0	0	2	7	0	0
*Charops* sp	3	6	0	0	0	0
*Telenomus* sp	1	2	0	0	0	0
*Trichogramma* sp	0	0	1	4	0	0
Total	51		28		6	
Diversity index (Shannon index)	0.2		0.1		0.0	
Species richness	8		7		4	

#### 3.2.4 Abundance, diversity, and richness of fall armyworm predators

The predator species diversity (0.9) and richness (3) were high in AER I and IIa. *Rhynocoris segmentarius* were the most abundant in AER IIa (57) followed by AER I (50). However, it was the lowest (1) in AER III (**Table 10**). *Belanogaster* sp was the most abundant in AER III. *Glypsus conspicuus* was found only in AER I and IIa (**Table 10**).

**Table 10:** Relative abundance, diversity and richness of fall armyworm predator species in Agroecological regions I, IIa and III in Zambia.

Predator	Agroecological region					
	I		IIa		III	
	No. of individuals	Relative Abundance (%)	No. of individuals	Relative Abundance (%)	No. of individuals	Relative Abundance (%)
Heteroptera: *Glypsus conspicuus*	5	10	3	8.1	0	0
Hymenoptera: *Belanogaster* sp	20	40	13	35	5	83
Heteroptera: *Rhynocoris segmentarius*	25	50	21	57	1	17
Total	50		37		6	
Diversity index (Shannon index)	0.9		0.9		0.4	
Species richness	3		3		2	

#### 3.2.5 Relationships between the number of natural enemies and climatic factors

**Figure 3:**
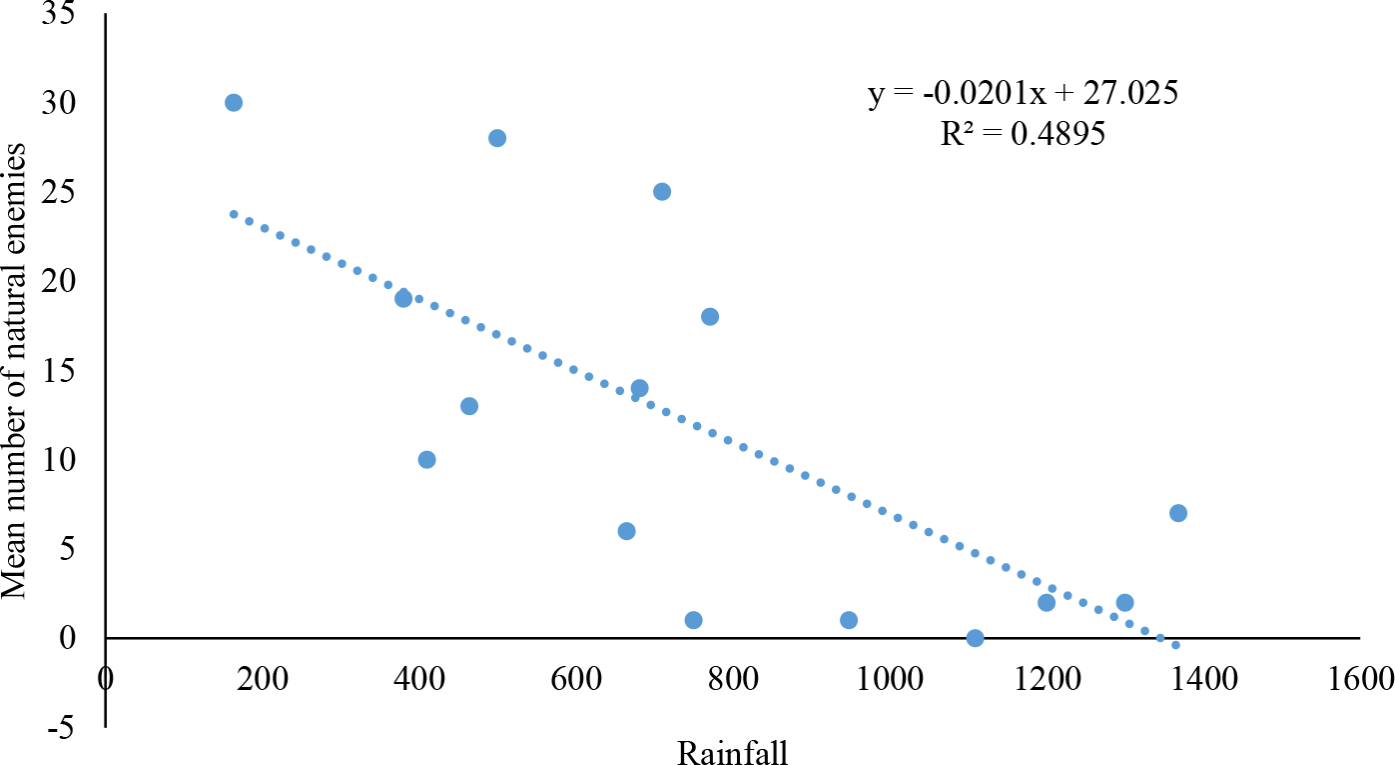
Relationship between fall armyworm natural enemies abundance and rainfall in the studied Agroecological regions of Zambia.

Significant differences were observed in the number of natural enemies collected in AERs in relation to rainfall (df= 1; P=0.004). A negative correlation was recorded between the number of natural enemies for FAW and rainfall (r^2 =^ 0.48) (**Figure 3**).

## 4 Discussions

A total of 11 FAW parasitoid species were collected and identified in this study, with Tachinid species, *Drino* species, and *D. quadrizonula* being the most abundant and widely distributed in all three AERs of Zambia. Similar findings have been reported by Shendange and Sathe (41) and Stireman et al. (42), who reported that Tachinid species occur worldwide and in nearly all terrestrial environments. Equally, in Argentina, Dipteran parasitoid species are reported to be FAW’s most widely dispersed biological control agents (43). Chinwada et al. (44) reported that Tachinids were also parasitoids for lepidopteran stemborers in Africa. In Nigeria, Tachinid species have been reported by Murua et al. (45) attacking *Spodoptera exampta* (Walker). In Ethiopia and Kenya, *Palexorista zonota* (Diptera: Tachinidae) was among the most-recorded larval parasitoids of FAW (25). In Mozambique, Caniço et al. (27) reported that Tachinid *D. quadrizonula* Thomson was among FAW’s most abundant larval parasitoids. The wide distribution could be explained by the wide array of lepidopteran pests serving as hosts for Tachinid species. Stemborers and other lepidopteran pests, such as the African armyworm *S. exampta*, are widely distributed in Zambia (10, 46). The arrival of FAW in Zambia has widened the host range of Tachinid species that lived on stemborers and African armyworm in the past.

The Tachinids were followed by egg–larval parasitoid *Chelonus* species, which were recorded from AERs I and IIa only. In the present study, *C. luteum* was the third most widely dispersed parasitoid and was recorded from all three AERs of Zambia, but in low numbers as compared with Tachinid and *Chelonus* species. It was more predominant in AERs I and IIa, which might be attributable to the high number of FAW recorded from those regions. In Tanzania and Kenya, *C. luteum* was the most common species (25) and, similarly, in Mozambique (27).

The absence of some parasitoid species in certain areas is attributed to the differences between locations, rainfall, maize crop stage, pest density and larval stage. Durocher-Granger et al. (26) reported similar factors influencing the occurrence of FAW parasitoids in Zambia. In this study, we demonstrated a negative relationship between rainfall and the number of natural enemies recorded from AERs. Although climatic factors are critical, the absence of *Chelonus* in AER III could also be due to the low numbers of FAW found in that region. Similar observations were reported in Ghana, where *Chelonus* sp, the most abundant parasitoid species, was obtained from 7 of 10 AERs (47).

Despite the coverage, egg parasitism was only observed in AERs I and IIa, while larval parasitism was recorded in all three regions. Egg parasitism by *Telenomus* sp and *Trichogramma* sp was very low in AERs I and IIa. Koffi et al. (47) reported an identical parasitism rate of 3.6% in a countrywide survey conducted in Ghana. Beserra and Parra (48)Dequech et al. (49), and Sun et al. (50) also reported similar observations. However, relatively higher results were reported in Mozambique (27) and in Uganda (51). The low egg parasitism could partly be explained by the fact that FAW covers its eggs with hairs and the short ovipositor of these small insects could not easily penetrate.

The low parasitism could also be attributed to the new associations of FAW and local parasitoids. FAW is a new pest in Africa (12, 52) and has an apparent association with indigenous lepidopteran parasitoids such as *C. icipe*, recently described from *S. littoralis* in Kenya (53), *C. luteum*, other parasitoids belonging to the subfamily *Campopleginae* that make them less efficient. However, in the current study, *Drino* sp contributed parasitism of 0.87%, *C. luteum* 0.35%, *D. quadrizonula* 0.34%, and *Chelonus* sp 0.38%.

Two species *Tiphia* sp and *Micromeriella* sp with parasitism rates of 0.07% reported for the first time attacking FAW larvae in Africa. *Tiphia* belongs to the family Tiphiidae, which are solitary wasps whose parasitoids attack various beetle larvae, especially in the Scarabaeoidea superfamily. Rogers and Potter (54) reported that *Tiphid* species had 33–58% parasitism. Their low parasitism in FAW could result from the pest being new to the parasitoid, and it is envisaged to increase with time.

Hemipterans (Reduviidae and Pentatomidae) and Hymenopterans (Vespidae) were the most common predators found in AERs I and IIa. This could be attributable to favourable environmental conditions and sufficient food, which enhance their survivability. *Rhynocoris segmentarius* (Reduviidae) was the most occurring and prevalent predator species in all AERs, with a total number of 47 individuals, followed by Hymenopterans of the *Belanogaster* genus (Vespidae) with 35 individuals and was equally recorded in all studied regions. Species of the Pentatomidae family were the lowest, with only 8 individuals recorded. The collected species are polyphagous as they prey on several other orders of insect species, including lepidopterans, as reported by Sahayaraj et al. (2020) (55).

Among other predators that were observed in the field and which have been reported in previous studies (56–58) but which were not found predating on FAW larvae or eggs were Hemiptera: stink bugs of unidentified species, Dermaptera: Forficulidae sp – earwig, Neuroptera: Chrysopidae: *Chrysoperla* sp and Coleoptera: Coccinellidae – ladybird beetles.

However, environmental factors and farming practices may also play an important role. Murua et al. (45) attributed low parasitism rates to temperature and rainfall, which are climatologic factors that have diverse effects on the density of the pest. During vegetative growth, the whorl of maize plants forms a funnel in which FAW larvae feed. The funnels collect water during heavy rains leading to drowning or dislodgement of FAW larvae. Similar observations were reported by Karthik et al. (59) for *Plutella xylostella*, where intense raindrops in the leaf axils dislodged newly hatched larvae from the plant, and a high proportion were killed by high precipitation. Prolonged periods and high incidence of rains in AER III could have caused low recovery rates natural enemies. The rainfall recorded in the 2018/19 season ranged from 380.4 mm in AER I to 1368.3 mm in AER III. Allen and Smith (60) reported a similar observation for *Cotesia medicaginis*, which reached its maximum longevity at 55% relative humidity, although longevity decreased markedly at levels above and below this value, of which the number of reproduction circles were reduced per year.

The higher diversity of natural enemies in AERs I and IIa could be attributed to high number of maize fields intercropped with legumes, pumpkins, and squash. Farmers intercropped maize with legumes throughout the year under rainfed and irrigation from Kariba dam, Luangwa and Zambezi rivers during the dry season. The intercropped could have high parasitoid and predator species diversity throughout the year. This is similar to the findings of Altieri et al. (61), who reported that when plant complexity increases in the agroecosystem through intercropping, cover crop and living mulch, the diversity of insects, including parasitoids and predator species, increases. The findings in our study in the intercrop and polycrops in AERs I and IIa are similar to observations by Khan et al. (62). Furthermore, Hind and Hooks (63) stated that increasing the flora complexity of agricultural habitats increased the survival and reproduction of natural enemies that promoted the biological control of FAW.

The absence or low occurrence of the predatory insects in AER III is associated with the absence of the outbreak of lepidopteran species, such as *Spodoptera exempta* Walker*, Eldana saccharina* Walker, *Chilo partellus* Swinhoe and *Busseola fusca* Fuller that have been reported in AERs I and IIa (64). Furthermore, the absence of a specific combination of crop structure and diversity in AER III could have contributed to the absence of the predatory bug. Mata et al. (65) stated that the effect of plant diversity is distinctly species-specific, with some species showing positive and others negative responses to trees, shrubs and crops.

Some of the fields surveyed were being sprayed with insecticides. This was affirmed through observations of containers and packaging materials for insecticides, such as Cypermethrine and Emamectin benzoate, which were poorly disposed in the fields in Kafue, Mkushi, Solwezi, and Mansa districts. In Zambia, insecticides are the first line of control strategy against FAW. In 2017, the Government of Zambia spent 3 million USD on the chemical control of FAW (15). Blanco et al. (66) and Tambo et al. (67) reported that, generally, control of FAW is usually achieved through the application of synthetic insecticides. Similar findings were reported by Kansiime et al. (68), that 60% of the farmers used insecticides to control FAW in Zambia. Therefore, the application of insecticides to control FAW could have contributed to the low parasitism percentage of the FAW in Zambia. Koffi et al. (47) reported that those fields that were not sprayed with insecticides had the highest parasitism percentages of 60% in Agogo Aburkyi, 55.6% in Legon, 33.3% in Kpong and 23.8% in Sanga in Ghana.

## 5 Conclusion

The study showed that natural enemies, including egg, egg-larval, and larval parasitoids, and predators are present in Zambia. Tachinid *Drino* species and *C. luteum*, *R. segmentarius* and *Belanogaster* sp are the most abundant and occurring FAW natural enemies. Egg parasitism is found in AER I and AER IIa while larval parasitism is found in all three surveyed regions. Variations in rainfall patterns affecting FAW availability and cropping systems in the three AERs may explain the differences natural enemies’ species diversity in Zambia. The FAW is a serious pest threatening cereal production in Zambia and information provided in this study can aid the development of a national biological control programme for its sustainable management.

## Data availability statement

The original contributions presented in the study are included in the article/supplementary materials. Further inquiries can be directed to the corresponding authors.

## Author contributions

GC, SP, PK and TT conceptualized. GC conducted the survey, laboratory experiments and wrote the original draft. GC and RC conducted morphological identification of natural enemies. SS, LO, FK and NS conducted molecular identification. GC, PS, PC, SN and SS revised the manuscript and approved the final draft. All authors contributed to the article and approved the submitted version.
